# Importance of Appropriate Counselling in Reducing Early Discontinuation of Norplant in a Northern District of Bangladesh

**Published:** 2014-03

**Authors:** Fauzia Akhter Huda, Sabiha Chowdhuri, Mohammad Fazla Rabbi Sirajuddin

**Affiliations:** ^1^icddr,b, GPO Box 128, Dhaka 1000, Bangladesh; ^2^Shaheed Suhrawardy Medical College & Hospital, Sher-e-Bangla Nagar, Dhaka 1207, Bangladesh

**Keywords:** Counselling, Decision-making, Informed choice, Norplant, Bangladesh

## Abstract

Bangladesh has achieved a considerable decline in fertility level in the past four decades through a strong family planning programme in spite of its poor social and economic growth. However, discontinuation of contraceptive methods and decline in the use of long-acting and permanent methods (LAPMs) are still the major concerns of the family planning programmes. This paper describes various factors that lead to the acceptance of the long-term method—Norplant and those that lead to early discontinuation. This descriptive, cross-sectional study was conducted in the Family Planning Association of Bangladesh (FPAB), Dinajpur, during March–June 2005 among 73 women who visited the clinic to remove their Norplant before its usual duration (5 years). The women were in the 25-31 years age-group—around two-thirds of them (57.5%) without formal education, and three-fourths of them (75.3%) were housewives. Most of them had been married for nine years, on average, and had two and/or more children. Sixty-seven percent of the women experienced regular menstruation, and 95% had used other contraceptive methods prior to using Norplant. Past users of Norplant were the single-most important source of information for about three-fourths of the women (74%); half of the women (51%) had discussed the method with their husbands, and majority (96%) of the husbands were informed about the women's decision on accepting the method before its implantation. All women were aware about the usual length of the effectiveness of Norplant. The most common reason for early removal of Norplant was menstrual disorder (59%), followed by desire for children (16%), husband's death, for abandonment or residing abroad (8%), anorexia, nausea, vomiting (7%), weight gain (4%), husband's objection (3%), and religious beliefs (3%). Service providers should properly counsel the couple before providing any contraceptive method, informing them about method-related side-effects and clearing any religious misconceptions. They should also explore the perception of women as well as their partners’ desire for children; couples who would like to have a baby within a year or two can be encouraged to use a short-term method that can be more easily discontinued.

## INTRODUCTION

Bangladesh has achieved a seven-fold increase in contraceptive-use in four decades from a mere 8% in 1975 to 61% in 2011. This contributed, to a large extent, in the decline of total fertility rate (TFR) from 6.3 in the 1970s to 2.3 in 2011 (1). How this country's family planning programme brought about such a dramatic decline in fertility despite poor social and economic growth has been the interest of many population scientists in the last decade. Hence, they have been keen to identify the critical features that make family planning services effective in meeting demand (2). Furthermore, the method mix has also changed over the last 20 years. The use of long-acting permanent methods (LAPMs) started to decline from the early 1990s (11% in 1993-1994 to 7.2% in 2004), stabilized in 2007 (7.3%), and slightly increased in 2011 (1,3). Currently, only 8% of the married couples use LAPMs, namely sterilization, intrauterine device (IUD), and implants, accounting for 13% of all contraceptive-usage. Comparatively, the use of LAPMs was much higher (12%) in 1991 and accounted for 30% of contraceptive-use. Between 2004 and 2011, there has been a slow increase in the use of male sterilization (0.6% to 1.2%) and implants (0.8% to 1.1%), although the use of these two methods lies at a very low rate of about 1%. This plateau in the use of long-term female methods is a cause for concern as fertility is now so low that most childbearing is completed by the mid- to late-twenties, and women have another two decades of reproductive life to protect themselves from unwanted pregnancies (1). Abortion ratios were found to be the highest among older women (in their thirties); thus it indicates that the women in this age-group have probably completed their desired family-size and do not need an effective contraceptive method to protect themselves from unwanted pregnancies (4).

The effectiveness of Norplant in preventing pregnancy is about 99%, although this figure may decrease with time. Since the drug is placed inside the woman's body, it does not have a high ‘typical’ (or user) failure rate (5,6). It is especially effective in the developing world as it does not require daily administration or visit to a hospital to be effective. In addition, no continual contraceptive supplies (pills, condoms, etc.) are necessary, and it is a highly-effective, low-cost contraceptive over the long term. An acceptability study of Norplant in 1987 showed that both continuers and discontinuers were satisfied with the method due to its long duration of action, efficacy, and convenience (7). Hence, increasing its use in the method mix has potential, given the current goal of the Government to increase the use of long-acting methods.

Discontinuation of family planning methods and reasons for such discontinuation remain a major concern for family planning programmes (1). About one in three users of contraceptive methods in Bangladesh stops using the method within 12 months after starting. The all-method discontinuation rate fluctuated between 47% and 49% during 1993 to 2004. In 2007, it increased to 57% and then sharply declined to 36% in 2011 (1).

The goals of family planning vary from couple to couple. Some women and men wish to protect fertility and health for future childbearing while others wish to prevent pregnancy entirely. Some seek family planning care to optimally space pregnancies; many wish to avoid any further pregnancies. One essential goal is optimal health and outcome—for both mother and baby—when pregnancy does occur (8). Concurrently, the family planning field has re-articulated its commitment to individuals’ and couples’ right to make voluntary choices about the number and timing of the childbirth they want and select compatible means to achieve their goals (2).

A woman's choice of contraceptive is influenced by many factors, and her requirements will change with time. These will reflect not only her social circumstances but also her medical condition (9). The concept of informed choice in decision-making on contraceptives requires that individuals or couples have effective access to a range of contraceptive options, adequate information about these options, and the ability to actively participate in decisions about their use (10). Research has shown that women have generally chosen a method before they seek medical advice and, unfortunately, that choices will, in most cases, have been made using information from the popular media and from friends or relatives. This inevitably leads to a negative bias, particularly in the case of long-term hormonal methods (11,12).

The aim of the present study was to analyze the factors that influence women's decisions to accept Norplant and to identify reasons for its early discontinuation.

## MATERIALS AND METHODS

### Study area

**Figure 1.**
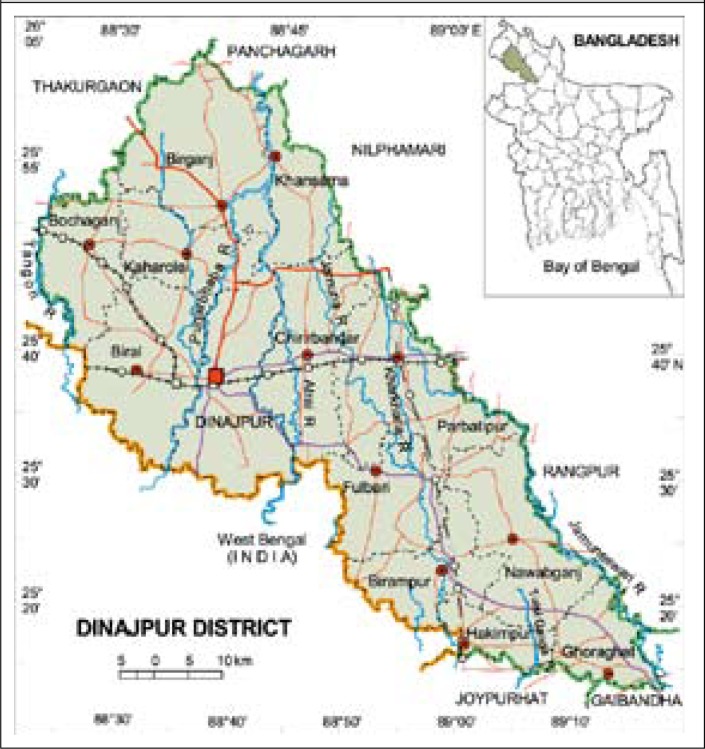
Map of Dinajpur district, Bangladesh

We conducted this descriptive, cross-sectional study in the Family Planning Association of Bangladesh (FPAB), Dinajpur, a small district in northern Bangladesh (Figure 1). The study was approved by Ethics Review Committee of NIPSOM.

The population of the study area is 2,642,850; among them, 51.60% are male, and 48.40% are female (13). The major source of income is agriculture, and average literacy rate is 45.67%, of which literacy rate among male is 51.02%, and that among female is 39.99% (13).

### Study population

We targeted all women who came to remove their Norplant earlier than its usual duration. Those women who provided informed consent to take part in this procedure were enrolled in the study. There were no exclusion criteria as the sample was drawn from all women undergoing early Norplant removal.

### Definition of early Norplant removal

Information on early removal of Norplant was collected for all women who had come to the family planning clinic to remove their Norplant before its usual duration (5 years). The term ‘early removal’ was used in this study to mean removal of Norplant within three years of its insertion.

### Data collection

All women attending the family planning clinic for early removal of Norplant during March 2005 to June 2005 were approached as they waited for the procedure and were informed about the study objectives. Women who agreed to participate in the study and provided informed consent were interviewed by means of a structured questionnaire for about 30-45 minutes. All the women were assured about receiving normal healthcare from the clinic if they even declined to participate in the study. We coordinated the timing of our approach to women with the care provider to ensure the most appropriate time for the interview, usually before going for the procedure. We also worked with the care provider and the facility to create an area of privacy for women when the survey was being carried out. All variables were organized into five main categories, and data were collected in the following areas: sociodemographic characteristics, menstrual history, reproductive history, knowledge about various family planning methods, factors influencing the decision to take Norplant and reasons for early discontinuation.

### Analysis of data

Data were compiled and edited meticulously by thorough checking and re-checking. All omissions and inconsistencies were corrected and methodically removed. Analysis was done by Statistical Package for Social Sciences (SPSS), (version ll.5) for Windows.

## RESULTS

### Description of the sample

During the four months of data-collection period, 97 women, who had their Norplant removed within three years of its insertion, were approached for participation in the study, and finally, 73 of them who agreed and provided informed consent were enrolled and interviewed.

### Sociodemographic characteristics of the study women

Majority (60%) of the women were between 25 and 29 years of age. Around two-thirds of the women (57.5%) had no formal education, and three-fourths of them (75.3%) were housewives. Most of them were from low-income families, and their average monthly income was Tk 4,000 (US$ 51). The mean marital age of the women was 9 years.

### Reproductive history and knowledge on family planning

Two-thirds of the women experienced a regular menstrual cycle, and one-third had irregular menstruation. All women had two or more children. Ninety-five percent of the women had used any other contraceptive method prior to Norplant insertion. Past users of Norplant were the single-most important sources of information for about three-quarters of the women (74%), and the second-most common sources of information were family planning workers (21%) (Table 1). All women were aware about the usual length of the effectiveness of Norplant (5 years). The advantage most-frequently mentioned by the service providers to the women was its length of effectiveness (89%) while only 42% of the women were informed by the providers about all disadvantages of the method. Side-effects of Norplant include: irregular menstrual bleeding, including prolonged menstrual bleeding during the first months of use (rarely heavy bleeding); untimely bleeding or spotting between periods; or no bleeding at all for several months. Other side-effects which may occur due to the use of hormonal methods include: headache, dizziness, nervousness, anxiety, nausea, and vomiting, adnexal enlargement, itching/rashes, acne, change of appetite, weight gain, breast tenderness, excessive hair growth, hair loss, and discolouration of the skin at the insertion site (http://www.popcouncil.org/what/norplant.asp).

**Table 1.** Source of information on Norplant

Source	Percentage
Norplant-users	74.0
Family planning workers	21.9
Doctors	4.1
Total	100.0

### Factors leading to the insertion of Norplant and its early removal

**Figure 2.**
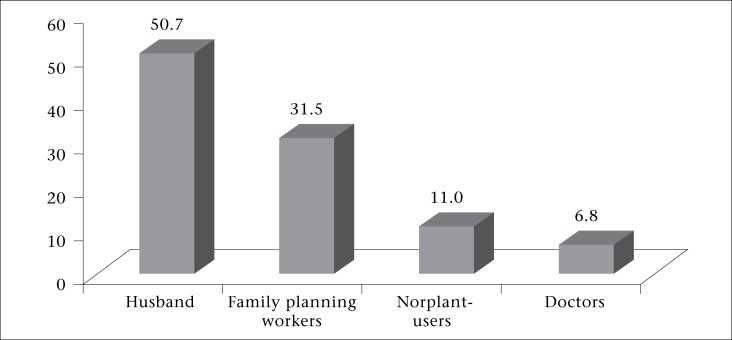
Distribution of women by discussant for Norplant insertion

Half of the women (51%) had a discussion with their husbands about the method prior to its insertion; 31% discussed with family planning workers, 11% with other Norplant-users, and the rest 7% with the medical doctors (Figure 2). The majority (96%) of husbands knew about their wives’ acceptance of Norplant before insertion while the remaining 4% were informed after insertion of the method. The most common reason for early removal of Norplant was menstrual disorder (59%), followed by desire for children (16%); husband's death, abandonment, or residing abroad (8%); anorexia, nausea, vomiting (7%); weight gain (4%); husband's objection (3%); and religious beliefs (3%) (Table 2).

**Table 2.** Distribution of women by cause of Norplant removal

Cause of removal	Number	Percentage
Menstrual disorder	43	58.9
Desire for child	12	16.4
Husband died/abandoned/abroad	06	8.2
Anorexia, nausea, vomiting	05	6.8
Weight gain	03	4.1
Husban's objection	02	2.7
Religious belief	02	2.7
Total	73	100.0

## DISCUSSION

Source of information on family planning methods plays an important role in the promotion and maintenance of contraceptive-use levels in the population. In an acceptability study of Norplant in Bangladesh, continuers were satisfied with the counselling they received before insertion while discontinuers did not (7). However, in our study, past users were the predominant sources of information on Norplant for our respondents. This indicates that, while it is important to spread information about availability via family members and friends in order to sustain the use, proper counselling by a care provider is important prior to insertion. The results of this study indicate that one-third of the women obtained information on the method from family planning workers. The decision to accept the implant is likely to have been influenced or supported mostly by the clinic staff of the aforementioned centre since the women are motivated to attend the clinic to obtain their method of choice (14). Contrary to the expectation that clinic workers would introduce Norplant to potential acceptors, past Norplant-users were the single-most, predominant, and important sources of information on the method while family planning workers tend to be a secondary source. Multiple responses have been given by some respondents and, thus, many may have obtained information on Norplant from more than one source, i.e. both past Norplant-users and FP workers. It is, therefore, indicative that verbal communication acted as one of the most important media in spreading the information on this long-term contraceptive method in Bangladesh (15-17).

Husband-wife communication about family planning and agreement to use contraceptive is not only necessary for adoption of certain methods, its absence might cause a serious impediment to using any of the methods. In Bangladesh, men often give their wives permission to practise contraception in a non-committal way, without actively supporting the decision themselves; if anything goes wrong, they can blame their wives. Men have the authority within the family but often they are reluctant to take the responsibility. Excellent inter-spousal communication is, therefore, an important intermediate step along the path towards eventual adoption and sustained use of contraceptive methods. Lack of discussion may reflect a lack of personal interest, hostility to the subject, or a customary reticence in talking about sex-related matters. It is particularly important for women who plan to use the method without their partner's knowledge or in defiance of his wishes to consider the side-effects, like menstrual irregularities that are likely with Norplant and how they will manage these side-effects in the light of family opposition (18,19). Two women in the present study had removed their Norplant due to husband's objection. Their Norplant removal took place close to the three-year cutoff point as set in our definition of early removal. These men were not aware of the method prior to its insertion into their wives’ body. Counselling of husbands prior to insertion of Norplant may have lowered discontinuation rates for menstrual disturbances, other medical reasons, personal reasons, and side-effects; according to a study in Bangladesh, male involvement in counselling is important in reducing discontinuation rates (12).

Discontinuation of family planning methods and reasons for such discontinuation remain a major concern for family planning programmes. All women in this study were well-aware that the implant provides protection from pregnancy for up to five years. Findings from the present study show that most of them chose this particular method considering its longer duration of effectiveness. It was found that a greater proportion of the women (59%) had removed Norplant due to experiencing menstrual disorder. This included prolonged or heavy bleeding as well as spotting. The changes in the menstrual pattern do not affect the women's health absolutely but irregular (and, therefore, unpredictable) or prolonged bleeding is significant because women need to make arrangements for their sanitary protection. Furthermore, irregular and prolonged bleeding affects both conjugal relations and religious practice. During menstruation, women are considered ritually impure. Thus, irregular and prolonged bleeding ultimately creates psychological problems among the users. A study of 1,570 Norplant-users in Indonesia found that women who had received counselling and information on Norplant were more satisfied than those who had received less information (20). The appropriate management of prolonged and excessive bleeding in Norplant-users is a key issue in reproductive health services. Considering the lack of an effective medical treatment for these problems, counselling before and throughout Norplant-use is of particular importance (21) Also, a study in Senegal showed that women who had been counselled about the full range of the side-effects of Norplant appeared abler to tolerate these side-effects and to seek treatment. In contrast, women who were not well-counselled about side-effects were often surprised and even frightened by their occurrence. Hence, counselling should include provision of detailed information on all possible side-effects to ensure continuation of method (22).

The second-most reported cause of removal was desire for children (16%). This factor is found to be an important predictor of implant discontinuation, which is often overlooked during counselling a woman before providing a contraceptive method. The counsellor should review not only the woman's reproductive intentions but also her partner's desire before providing any contraceptive method, especially a long-term method, like Norplant. If women and/or their husbands would like to get another child within one year or two, they could be encouraged to use a method that can be more easily discontinued. It is possible that some women may choose not to use a long-term method after such counselling (23,24).

### Limitations

This study had a number of limitations, such as small sample-size, shorter duration, limited to only one family planning centre, and old age of the data. Also, recollections about the original decision-making for the use of Norplant were derived from recall of events that happened 3 years earlier. Thus, there may be a recall bias. Given the lack of shorter duration of the study, there was no way to accurately describe the situation of discontinuation of Norplant earlier in other parts of the country. The estimate for earlier Norplant removal reported in this study is, thus, conservative. The definition of ‘early Norplant removal’ used in the study was particularly specific for this study. Therefore, some possible misclassification might happen as there is no existing standard guideline for defining the early removal of the method. The old age of the data is another important limitation of the study but one of the drivers of the Program Implementation Plan (PIP) of the new Health, Population and Nutrition Sector Development Program (HPNSDP) 2011-2016 include addressing population growth by means of a vigorous fully-integrated family planning services, with a focus on strengthening the service in long-acting and permanent methods (LAPMs). Under this component, one of the activities includes reducing dropout of long-acting methods. Findings from this study will complement this activity by providing the reasons behind dropout rates. Hence, although these data are one decade old, these are still relevant to the current activities of HPNSDP 2011-2016.

### Conclusions

A very effective communication environment needs to be created credibly between healthcare providers and contraceptive-users. Before providing any contraceptive method, counselling should be done in a way that enables women to fully evaluate the strengths and weaknesses of their contraceptive options so that they can ultimately make free and informed choices, along with their partners’ involvement, which may lead to a significant reduction in the total discontinuation rates of long-term contraceptive methods. Proper counselling about side-effects and their early management as well as clearing religious misconceptions are necessary to reduce the rate of early Norplant removal. Service providers should also discuss with couples about their desire for children, and, if the couple wishes to have a baby within a year or two, they can be encouraged to use a short-term method that can be more easily discontinued.

## ACKNOWLEDGEMENTS

The project team would like to express their gratitude to the valued members of the Family Planning Association of Bangladesh (FPAB), Dinajpur, for their generous support. The team members are grateful to all the study participants; without their assistance and cooperation, it could not be possible to conduct the study.
